# Author Correction: The rate and molecular spectrum of mutation are selectively maintained in yeast

**DOI:** 10.1038/s41467-023-37354-7

**Published:** 2023-04-04

**Authors:** Haoxuan Liu, Jianzhi Zhang

**Affiliations:** Department of Ecology and Evolutionary Biology, University of Michigan, Ann Arbor, MI USA

**Keywords:** Evolutionary genetics, Mutation

Correction to: *Nature Communications* 10.1038/s41467-021-24364-6, published online 30 June 2021

In the original version of this Article, some mutation accumulation (MA) lines did not have the correct *MSH2* inserted; instead, the inserted *MSH2* lacked the stop codon and encoded 24 extra amino acids at the 3’ end. Specifically, three of the 13 original second-order MA lines (MA28, MA33, and MA38) had the correct *MSH2* inserted, while the remaining 10 had the *MSH2* that lacked the stop codon. To correct this error, the wild-type *MSH2* was inserted into all lines after the first round of MA, the inserted *MSH2* sequence was confirmed by Sanger sequencing, and the downstream experiments were repeated, including fluctuation test, second round of MA, and whole-genome sequencing after the second round of MA. The newly generated data have been reanalyzed and presented in the corrected version of this Article. The following changes have been made:

- In the Results subsection ‘Mutagenesis frequently reduces μ’, the third and fourth paragraphs have been modified from:
  “Because the *N*_*e*_ of the MA lines was about 10 (see “Methods”) while most mutations are expected to have a fitness effect on the order of 1% or smaller^21^, selection should be infrequent during the MA and was indeed the case (see “Methods”). To assess the impact of MA on μ, we first inserted *MSH2* back to the MA lines, which was accomplished in 66 of the 93 lines (Fig. 1d). Using the classic fluctuation test based on the reporter gene *CAN1* (see “Methods”), we successfully measured μ in the progenitor as well as 48 of the above 66 MA lines, all carrying an intact *MSH2* (Fig. 1d). We found μ of the 48 MA lines to range from 0.01 to 27 times that of the progenitor (Fig. 2a, Supplementary Data 1), including 28 lines with significantly higher μ and 12 lines with significantly lower μ than the progenitor (see “Methods”). Furthermore, 10 of the 12 lines with significantly decreased μ had μ reduced by at least 50%, while the remaining two lines had μ reduced by 46% and 38%, respectively (Fig. 2a). That 30% of MA lines with significantly altered μ exhibit such drastic reductions in μ is inconsistent with the DBH, because when μ is near the drift barrier, mutations are expected to be strongly biased toward increasing μ and are not expected to cause such large reductions of μ so frequently (Fig. 1b). Our finding suggests that the progenitor’s μ is well above the drift barrier (Fig. 1c). We found no significant correlation between μ and the number of mutations accumulated during the MA or the growth rate of the MA line (Supplementary Fig. 2).
  Because the above estimation of μ was based on loss-of-function mutations in one gene, we attempted to verify these results by performing another round of MA followed by WGS in 13 of the above 48 MA lines as well as the progenitor, all with an intact *MSH2* (Fig. 1d). 18–20 parallel lines were established from each strain, and on average 695 generations of MA were performed in the medium similar to that used in the fluctuation test (Supplementary Table 2, Supplementary Data 1, see “Methods”). The MA + WGS results were generally consistent with those from the fluctuation test. For instance, compared with the progenitor, all five lines with higher *CAN1*-based μ exhibited higher MA+ WGS-based SNV (Fig. 2b) and indel (Fig. 2c) rates. Among the eight lines with lower *CAN1*-based μ, four exhibited significantly lower MA + WGS-based SNV and/or indel rates; the reduced consistency here is likely due to larger errors of lower *CAN1*-based μ estimates (Fig. 2a). *CAN1*-based μ is significantly correlated with the MA + WGS-based SNV rate (*r* = 0.64, *P* = 0.013; Fig. 2d) but not with the MA+ WGS-based indel rate (*r* = 0.11, *P* = 0.70; Fig. 2e). This observation is not unexpected given that most loss-of-function mutations in *CAN1* are SNVs instead of indels^22^.”
  to now read:
  “Because the *N*_*e*_ of the MA lines was about 10 (see “Methods”) while most mutations are expected to have a fitness effect on the order of 1% or smaller^21^, selection should be infrequent during the MA and was indeed the case (see “Methods”). To assess the impact of MA on μ, we first inserted *MSH2* back to the MA lines, which was accomplished in 60 of the 93 lines (Fig. 1d). Using the classic fluctuation test based on the reporter gene *CAN1* (see “Methods”), we successfully measured μ in the progenitor as well as 49 of the above 60 MA lines, all carrying an intact *MSH2* (Fig. 1d). We subsequently found that five of the 49 lines were likely diploidized upon the insertion of *MSH2* (see the following paragraph) and excluded them (marked with stars in Fig. 2a) from all *CAN1*-based analyses. We found μ of the 44 remaining MA lines to range from 0.01 to 26 times that of the progenitor (Fig. 2a, Supplementary Data 1), including 19 lines with significantly higher μ and 13 lines with significantly lower μ than the progenitor (see “Methods”). Furthermore, 10 of the 13 lines with significantly decreased μ had μ reduced by at least 50%, while the remaining three lines had μ reduced by 40% to 43% (Fig. 2a). That over 40% of MA lines with significantly altered μ exhibit such drastic reductions in μ is inconsistent with the DBH, because when μ is near the drift barrier, mutations are expected to be strongly biased toward increasing μ and are not expected to cause such large reductions of μ so frequently (Fig. 1b). Our finding suggests that the progenitor’s μ is well above the drift barrier (Fig. 1c). We found a significant positive correlation between μ and the number of mutations accumulated during the MA, but μ was not significantly correlated with the growth rate of the MA line (Supplementary Fig. 2).
  Because the above estimation of μ was based on loss-of-function mutations in one gene, we attempted to verify these results by performing another round of MA followed by WGS in 16 of the above 49 MA lines as well as the progenitor (and a diploid version of the progenitor), all with an intact *MSH2* (Fig. 1d). 4–20 parallel lines were established from each strain, and on average 684 generations of MA were performed in the medium similar to that used in the fluctuation test (Supplementary Table 2, Supplementary Data 1, see “Methods”). Four of the 16 MA lines were apparently diploid, because the majority of the mutations observed in MA+WGS were in heterozygous state. Diploids should not produce mutant colonies in the fluctuation test. To be conservative in inferring mutation rate reductions in the first round of MA, we additionally regarded a line that was not subject to MA+WGS but had only two mutant colonies in the fluctuation test as putatively diploid (right most line marked with a star in Fig. 2a). We excluded these five diploid lines from all *CAN1*-based analyses. Because haploid and diploid progenitors showed similar mutation rates (Fig. 2b, c), all 16 lines with MA+WGS were included in analyses based solely on MA+WGS. The MA + WGS results were generally consistent with those from the fluctuation test. For instance, compared with the progenitor, all eight lines with higher *CAN1*-based μ exhibited higher MA+ WGS-based SNV (Fig. 2b) or indel (Fig. 2c) rates. Among the four lines with lower *CAN1*-based μ, three exhibited significantly lower MA + WGS-based SNV or indel rates; the reduced consistency here is likely due to larger errors of lower *CAN1*-based μ estimates (Fig. 2a). *CAN1*-based μ is significantly correlated with both the MA + WGS-based SNV rate (*r* = 0.88, *P* = 8.9×10^-5^; Fig. 2d) and the MA+ WGS-based indel rate (*r* = 0.78, *P* = 1.6×10^-3^; Fig. 2e), although the latter correlation is weaker than the former. This observation is not unexpected given that most loss-of-function mutations in *CAN1* are SNVs instead of indels^22^.”
- In the Results subsection ‘Stabilizing selection of μ’, the first paragraph sentence
  “To estimate *V*_m_ that is comparable with *V*_g_, we used the *CAN1*-based μ estimates from the 48 MA lines, but corrected for the increased mutagenesis in the MA induced by deleting *MSH2*.”
  has been corrected to read ‘44 haploid’ in place of ‘48’.
  The second paragraph has been modified from:
  “We found *V*_g_*/V*_m_ to be at least 800 times lower than the neutral expectation of 4*N*_*e*_ ≈ 4 × 10^7^ (see “Methods”), regardless of the particular *V*_m_ used (Table 1), indicating strong stabilizing selection of μ. This signal of stabilizing selection is not an artifact of the physical limits of μ, because the range of μ among the natural strains is even smaller than that of the MA lines (Fig. 2a). To investigate whether the stabilizing selection prohibits the evolution of higher μ, lower μ, or both, we separated *V*_m_ into two components that respectively measure the variance of μ created by mutations decreasing μ (*V*_mL_) and increasing μ (*V*_mH_). If there is no selection against a reduction in μ, *V*_g_ should be at least as large as 4*N*_*e*_*V*_mL_. However, we found *V*_g_ to be at least 700 times lower than 4*N*_*e*_*V*_mL_ (Table 1), indicating the action of selection prohibiting a reduction of μ in evolution. Similarly, *V*_g_ was at least 150 times lower than 4*N*_*e*_*V*_mH_ (Table 1), indicating the action of selection prohibiting a rise of μ in evolution. In the above tests, the smallest difference observed between *V*_g_ and a neutral expectation was 150 times, based on *V*_m2_ that corresponds to a conservative test. Therefore, it is exceedingly unlikely that our test results are due to confounding factors such as mutation spectrum differences between wild-type and *MSH2*-lacking strains or the inaccuracy of yeast’s *N*_*e*_ estimate (see “Methods”). Together, the above results demonstrate that μ has been selectively maintained at an intermediate level in *S. cerevisiae*.”
  to now read:
  “We found *V*_g_*/V*_m_ to be at least 540 times lower than the neutral expectation of 4*N*_*e*_ ≈ 4 × 10^7^ (see “Methods”), regardless of the particular *V*_m_ used (Table 1), indicating strong stabilizing selection of μ. This signal of stabilizing selection is not an artifact of the physical limits of μ, because the range of μ among the natural strains is even smaller than that of the MA lines (Fig. 2a). To investigate whether the stabilizing selection prohibits the evolution of higher μ, lower μ, or both, we separated *V*_m_ into two components that respectively measure the variance of μ created by mutations decreasing μ (*V*_mL_) and increasing μ (*V*_mH_). If there is no selection against a reduction in μ, *V*_g_ should be at least as large as 4*N*_*e*_*V*_mL_. However, we found *V*_g_ to be at least 300 times lower than 4*N*_*e*_*V*_mL_ (Table 1), indicating the action of selection prohibiting a reduction of μ in evolution. Similarly, *V*_g_ was at least 230 times lower than 4*N*_*e*_*V*_mH_ (Table 1), indicating the action of selection prohibiting a rise of μ in evolution. In the above tests, the smallest difference observed between *V*_g_ and a neutral expectation was 230 times, based on *V*_m2_ that corresponds to a conservative test. Therefore, it is exceedingly unlikely that our test results are due to confounding factors such as mutation spectrum differences between wild-type and *MSH2*-lacking strains or the inaccuracies of *V*_m_, *V*_g_, and *N*_e_ estimates (see “Methods”). Together, the above results demonstrate that μ has been selectively maintained at an intermediate level in *S. cerevisiae*. Note that the selective forces to increase and to suppress μ are not equally strong, because the mean μ of the MA lines is higher than the progenitor (*P* = 4.3 × 10^-3^ for *CAN1*-based μ, *t*-test; *P* = 5.7 × 10^-7^ for WGS-based SNV rate, *t*-test).”
  The third paragraph sentences:
  “We obtained *V*_m_ based on the 13 MA lines with MA+ WGS-based estimates of SNV rates and corrected the impact of deleting *MSH2* as in the above analysis. We found *D*^2^/*V*_m_ to be at least 10^4^ times smaller than *T* (Table 1). We also respectively estimated *V*_mL_ and *V*_mH_ using the 13 MA lines with MA+ WGS-based estimates of μ. Again, we found *D*^2^/*V*_mL_ to be at least 300 times smaller and *D*^2^/*V*_mH_ at least 6000 times smaller than *T* (Table 1), demonstrating selection against lowering as well as increasing μ in the divergence of *Saccharomyces* species.”
  have been modified to now read:
  “We obtained *V*_m_ based on the 16 MA lines with MA+ WGS-based estimates of SNV rates and corrected the impact of deleting *MSH2* as in the above analysis. We found *D*^2^/*V*_m_ to be at least 4000 times smaller than *T* (Table 1). We also respectively estimated *V*_mL_ and *V*_mH_ using the 16 MA lines with MA+ WGS-based estimates of μ. Again, we found *D*^2^/*V*_mL_ to be at least 400 times smaller and *D*^2^/*V*_mH_ at least 2400 times smaller than *T* (Table 1), demonstrating selection against lowering as well as increasing μ in the divergence of *Saccharomyces* species.”
- In the Results subsection ‘Discovery of *PSP2* as a mutator gene’, the first paragraph sentences:
  “To this end, we first identified candidate mutator genes by screening genes that were more frequently mutated in low-μ lines than in high-μ lines among the 48 MA lines with *CAN1*-based μ estimates (Supplementary Table 5). We then picked four candidates (*RAD9*, *YFL013W-A*, *PSP2*, and *MSH4*) based on their ranks from the screening and annotated functions for a follow-up study.”
  have been modified to now read:
  “We originally identified candidate mutator genes by screening genes that were more frequently mutated in low-μ lines than in high-μ lines among MA lines with *CAN1*-based μ estimates (Supplementary Table 5), and picked four candidates (*RAD9*, *YFL013W-A*, *PSP2*, and *MSH4*) based on their ranks from the screening and annotated functions for a follow-up study. We subsequently found that some MA lines had erroneous *MSH2*, so reinserted *MSH2* followed by re-estimation of *CAN1*-based μ. Based on these new estimates of μ (Fig. 2a), the four genes are ranked 1362, 911, 58, and 76, respectively.”
- In the Results subsection ‘Mutation spectrum has been shaped by selection’, the first paragraph sentences:
  “To investigate the potential role of natural selection in shaping yeast’s mutation spectrum, we compared the variance (*V*_g_) in a component of the mutation spectrum among five divergent natural yeast strains having published MA+WGS data (Supplementary Fig. 3), with the corresponding mutational variance per generation estimated from the 13 MA lines with MA+WGS data. For instance, even under the most generous calculation, *V*_g_/*V*_m_ (4.48 × 10^4^) of the proportion of mutations that are SNVs is orders of magnitude smaller than the neutral expectation of 4 × 10^7^ (Supplementary Table 6). In fact, the variance of the proportion of SNVs is smaller among the five natural strains than among the 13 MA lines (Fig. 4a), despite that the numbers of generations separating the natural strains are much greater than those separating the MA lines even after the correction for the increased mutagenesis of MA lines induced by deleting *MSH2*. Similar results were found regarding the proportion of insertions (maximal *V*_g_/*V*_m_=7.84 × 103) and that of deletions (maximal *V*_g_/*V*_m_=5.12 × 10^4^) (Fig. 4a, Supplementary Table 6).”
  have been modified to now read:
  “To investigate the potential role of natural selection in shaping yeast’s mutation spectrum, we compared the variance (*V*_g_) in a component of the mutation spectrum among five divergent natural yeast strains having published MA+WGS data (Supplementary Fig. 3), with the corresponding mutational variance per generation estimated from the 16 MA lines with MA+WGS data. Because haploid and diploid progenitors show similar mutational spectrums (Fig. 4), we analyze the MA lines and natural strains regardless of their ploidy. Even under the most generous calculation, *V*_g_/*V*_m_ (3.07 × 10^4^) of the proportion of mutations that are SNVs is orders of magnitude smaller than the neutral expectation of 4 × 10^7^ (Supplementary Table 6). In fact, the variance of the proportion of SNVs is smaller among the five natural strains than among the 16 MA lines (Fig. 4a), despite that the numbers of generations separating the natural strains are much greater than those separating the MA lines even after the correction for the increased mutagenesis of MA lines induced by deleting *MSH2*. Similar results were found regarding the proportion of insertions (maximal *V*_g_/*V*_m_= 3.57 × 103) and that of deletions (maximal *V*_g_/*V*_m_= 3.27 × 10^4^) (Fig. 4a, Supplementary Table 6).”
  The second paragraph sentence:
  “There are six different types of SNVs (Fig. 4b) and we found evidence for stabilizing selections on each of the six fractions (maximal *V*_g_/*V*_m_ ranging between 1.51 × 10^4^ and 1.31 × 10^6^; Supplementary Table 6).”
  has been corrected to read ‘3.03 × 10^4^’ in place of ‘1.51 × 10^4^’ and ‘1.35 × 10^6^’ in place of ‘1.31 × 10^6^’.
  The third paragraph sentences:
  “We found evidence for stabilizing selection of Ts/Tv (Fig. 4c); the maximal *V*_g_/*V*_m_ equals 4.11 × 10^4^ (Supplementary Table 6). In particular, Ts/Tv is higher than that of the progenitor in 10 of the 13 MA lines, and Ts/Tv is increased in all 7 MA lines with a significantly altered Ts/Tv (P = 0.016, two-tailed binomial test) (Fig. 4c).”
  have been modified to now read:
  “We found evidence for stabilizing selection of Ts/Tv (Fig. 4c); the maximal *V*_g_/*V*_m_ equals 9.13 × 10^4^ (Supplementary Table 6). In particular, Ts/Tv is significantly higher (or lower) than that of the progenitor in two (or zero) MA lines (Fig. 4c).”
  The fourth paragraph sentences:
  “We found that the ratio of the number of GC→AT mutations to the number of AT→GC mutations is significantly different in 7 of the 13 MA lines when compared with the progenitor, including 6 lines that increased the bias and one line that reversed the bias to the opposite direction (Fig. 4d). Clearly, the AT mutational bias is subject to genetic control and is not a chemical necessity. Furthermore, *V*_g_/*V*_m_ for the AT mutational bias is at least 700 times lower than the neutral expectation (Supplementary Table 6), indicating that the bias has been maintained by stabilizing selection.”
  have been modified to now read:
  “We found that, in one MA line, the ratio of the number of GC→AT mutations to the number of AT→GC mutations is significantly different from that in the progenitor, and is reversed from >1 to <1 (Fig. 4d). Clearly, the AT mutational bias is subject to genetic control and is not a chemical necessity. Furthermore, *V*_g_/*V*_m_ for the AT mutational bias is at least 120 times lower than the neutral expectation (Supplementary Table 6), indicating that the bias has been maintained by stabilizing selection.”
- In the Methods subsection ‘Strains and genetic manipulations’, the first paragraph sentences:
  “Transformation was performed in each MA line for up to three times. Restoration of *MSH2* was successful in only 66 of the 93 MA lines, probably due to reduced transformation efficiencies in the MA lines.”
  have been modified to now read:
  “Transformation was performed in each MA line for up to three times and was confirmed by Sanger sequencing of the reinserted locus. Restoration of *MSH2* was successful in only 60 of the 93 MA lines, probably due to reduced transformation efficiencies in the MA lines.”
- In the Methods subsection ‘MA and whole-genome sequencing’, the second paragraph sentences:
  “A total of 14 strains, including 13 of the above 93 MA lines and BY4741, all with intact *MSH2*, were subject to the second round of MA. Eighteen to 20 replicate lines were established for each strain. Cells were propagated at 30 °C on SC (synthetic complete) plates, similar to that used in the fluctuation test. The total time in the second round of MA for all lines was kept at ~100 days and the number of generations between bottlenecks was kept at ~20. The between-bottleneck duration was different among these 14 strains because of their different generation times. It was 48 h in BY4741, MA28, MA29, and MA38, 72 h in MA13, MA23, MA25, MA33, MA77, MA78, and MA85, and 96 h in MA45, MA59, and MA95. The genomes of 272 MA lines at the end of the second round of MA and their 14 ancestral strains were sequenced.”
  have been modified to now read:
  “A total of 18 strains, including 16 of the above 93 MA lines, BY4741 (haploid), and BY4743 (diploid), all with intact *MSH2*, were subject to the second round of MA. Four to 20 replicate lines were established for each strain. Cells were propagated at 30 °C on SC (synthetic complete) plates, similar to that used in the fluctuation test. The total time in the second round of MA for all lines was kept at ~100 days and the number of generations between bottlenecks was kept at ~20. The between-bottleneck duration was different among these 18 strains because of their different generation times. It was 48 h in BY4741, BY4743, MA28, and MA38, 72 h in MA15, MA21, MA23, MA25, MA29, MA33, MA44, MA63, and MA92, and 96 h in MA45, MA51, MA56, MA64, and MA94. The genomes of 209 MA lines at the end of the second round of MA and their 18 ancestral strains were sequenced.”
- In the Methods subsection ‘Fluctuation test’, the first paragraph sentence:
  “We subjected all 66 MA lines with reinserted *MSH2* to the fluctuation test, but only 48 of them grew in the medium.”
  has been corrected to read ‘60’ in place of ‘66’ and ‘49’ in place of ‘48’.
  The second paragraph sentence:
  “No *CAN1* mutant was observed in 4 MA lines, and their μ values were calculated by assuming the observation of one mutant colony to allow plotting μ in a logarithmic scale.”
  has been corrected to read ‘two’ in place of ‘4’.
- In the Methods subsection ‘Estimation of *V*_m_, *V*_g_, and *D*^2^’, a new paragraph was appended to the end:

“The *V*_g_ and *V*_m_ calculated here are phenotypic variances, including the genetic component and estimation error, because the environment is fixed. The phenotypic variance caused by estimation error should be similar for natural strains and MA lines because of the use of the same phenotyping method. Because the phenotypic variance is greater for MA lines than for natural strains, the fraction of phenotypic variance contributed by genetics is greater for MA lines than for natural strains. Hence, *V*_g_/*V*_m_ is overestimated when computed using phenotypic variance instead of genetic variance, which renders our conclusion that *V*_g_/*V*_m_ is smaller than the neutral expectation conservative.”

- In Figure 1d, the number of lines has been corrected.
- The previous incorrect version of Figure 2:

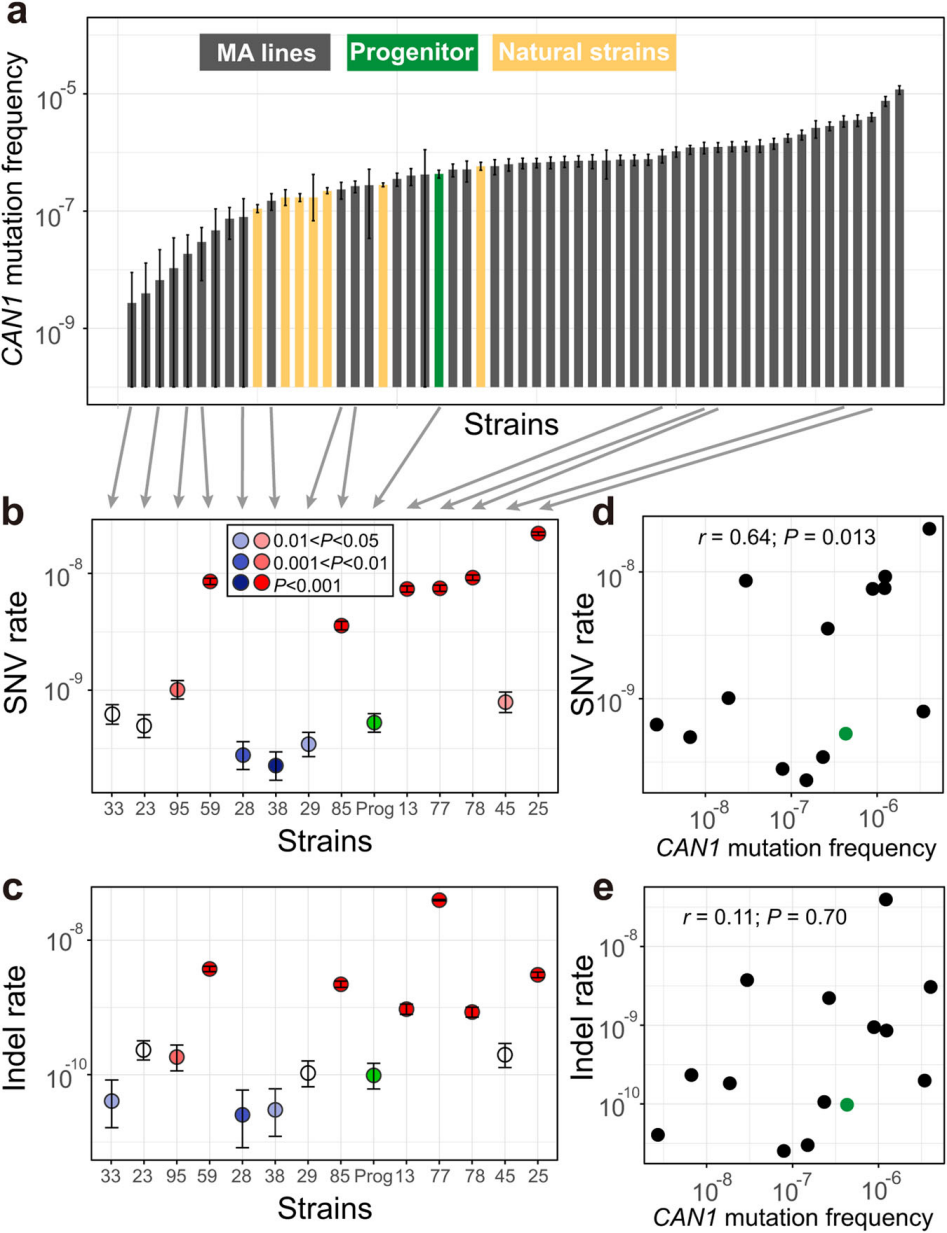


“**Fig. 2 Mutation frequencies and rates of the MA lines.** **a** *CAN1* mutation frequencies of the progenitor (green), 48 MA lines (gray), and 7 natural yeast strains (yellow), determined by the fluctuation test based on 72 (green and gray) or 288 (yellow) biologically independent cell cultures from each strain. The data from the seven natural strains came from ref. ^14^. Error bars indicate 95% confidence intervals of the mean. Mutation frequency is the probability of loss-of-function mutation in *CAN1* per cell division, so is not directly comparable with the mutation rates estimated by MA+ WGS. SNV (**b**) or indel (**c**) mutation rate per site per generation in the progenitor (green) and 13 MA lines estimated by MA+ WGS based on 18 to 20 biologically independent replicates. Numbers on the X-axis refer to IDs of MA lines, while “Prog” refers to the progenitor. Circles represent mean values while error bars show 95% confidence intervals predicted from Poisson distributions. Significant rate differences from the progenitor are indicated by blue (lower than the progenitor) or red (higher than the progenitor) with different shades for different nominal *P* values from Wilcoxon rank-sum tests. White circles show no significant rate difference from the progenitor. Correlation between SNV (**d**) or indel (**e**) mutation rate measured by MA+ WGS and CAN1 mutation frequency measured in fluctuation test among the 13 MA lines and the progenitor (green). Pearson’s *r* (based on the values before the log_10_-transformation) and associated *P* value are shown. The green dot shows the progenitor.”

has been replaced with the correct version of Figure 2
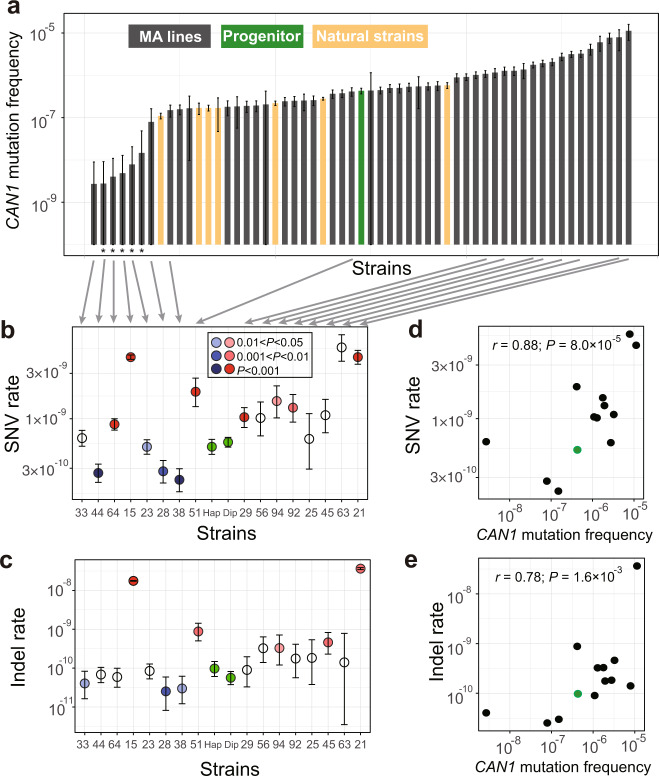


**Fig. 2 Mutation frequencies and rates of the MA lines.** **a** *CAN1* mutation frequencies of the progenitor (green), 49 MA lines (gray), and 7 natural yeast strains (yellow), determined by the fluctuation test based on 72 (green and gray) or 288 (yellow) biologically independent cell cultures from each strain. The data from the seven natural strains came from ref. ^14^. Error bars indicate 95% confidence intervals of the mean. “*” on the *X*-axis indicates diploid or putatively diploid strains. Mutation frequency is the probability of loss-of-function mutation in *CAN1* per cell division, so is not directly comparable with the mutation rates estimated by MA+ WGS. SNV (**b**) or indel (**c**) mutation rate per site per generation in the progenitor in both haploid and diploid form (green) and 16 MA lines estimated by MA+ WGS based on 4 to 20 biologically independent replicates. Numbers on the X-axis refer to IDs of MA lines, while “Hap” refers to the progenitor in the haploid form and “Dip” refers to the progenitor in the diploid form. Circles represent mean values while error bars show 95% confidence intervals predicted from Poisson distributions. Significant rate differences from the progenitor are indicated by blue (lower than the progenitor) or red (higher than the progenitor) with different shades for different nominal *P* values from Wilcoxon rank-sum tests. White circles show no significant rate difference from the progenitor. Correlation between SNV (**d**) or indel (**e**) mutation rate measured by MA+ WGS and CAN1 mutation frequency measured in fluctuation test among the 12 haploid MA lines and the haploid progenitor (green). Pearson’s *r* (based on the values before the log_10_-transformation) and associated *P* value are shown. The green dot shows the progenitor.

- The previous incorrect version of Figure 4:
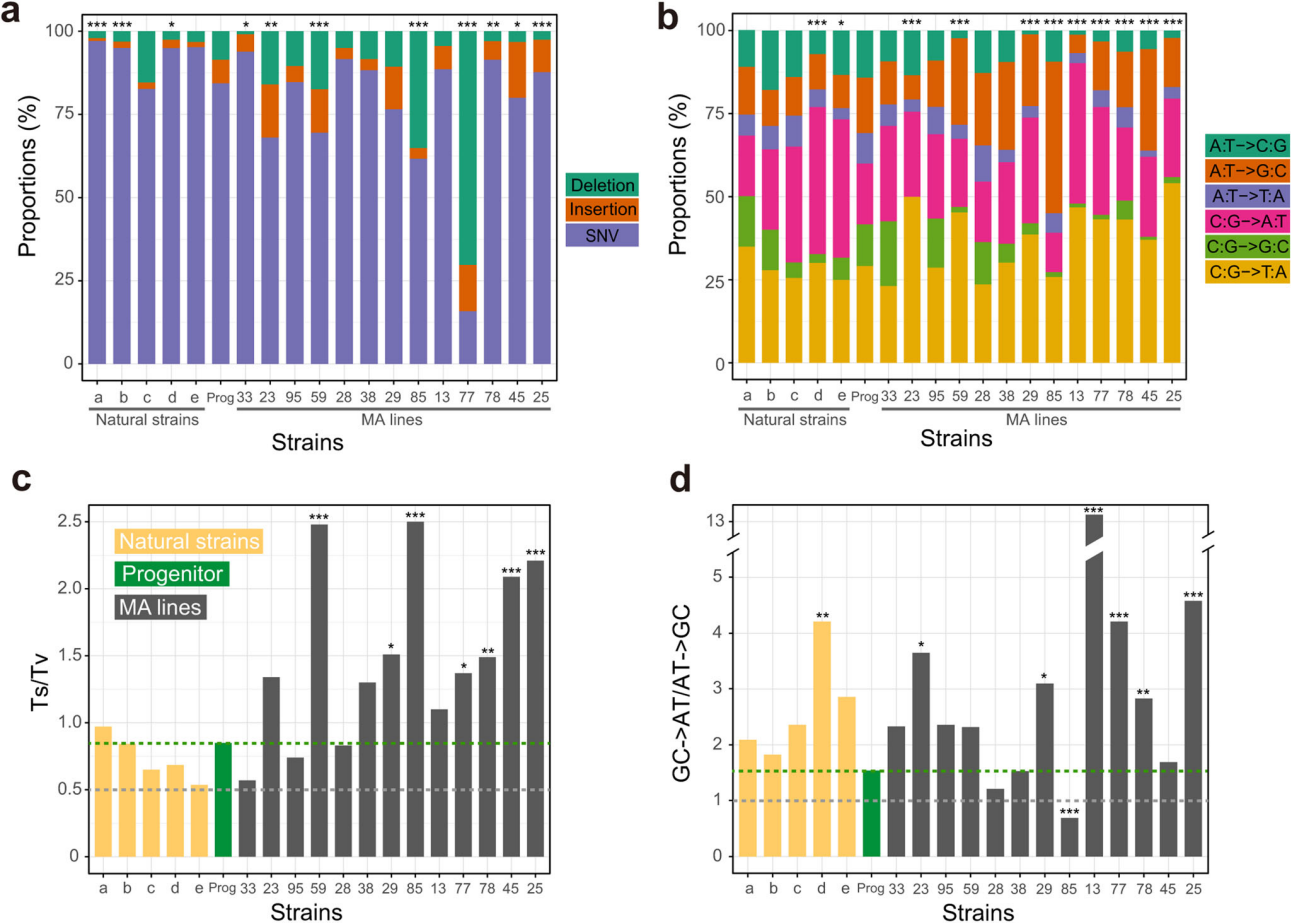

“**Fig. 4 Molecular spectra of mutations in 13 MA lines and 5 natural yeast strains estimated by MA + WGS.** **a** Proportions of SNVs, insertions, and deletions among all mutations. **b** Relative proportions of the six types of SNVs. **c** Number of transition mutations relative to the number of transversion mutations (Ts/Tv). **d** Number of GC→AT mutations relative to the number of AT→GC mutations. In **c** and **d**, the gray dotted line shows the random expectation while the green dotted line shows the value in the progenitor. In all panels, the small letters on the X-axis refer to natural strains (**a** DBY4974/DBY4975; **b** SEY6211; **c** SK1/BY; **d** DBVPG6765; and **e** YPS128/DBVPG6765), “Prog” refers to the progenitor, and the numbers refer to IDs of MA lines. Two-tailed chi-squared test is performed between each strain and the progenitor: *, P < 0.05; **, *P* < 0.01; ***, *P* < 0.001.”

has been replaced with the correct version of Figure 4
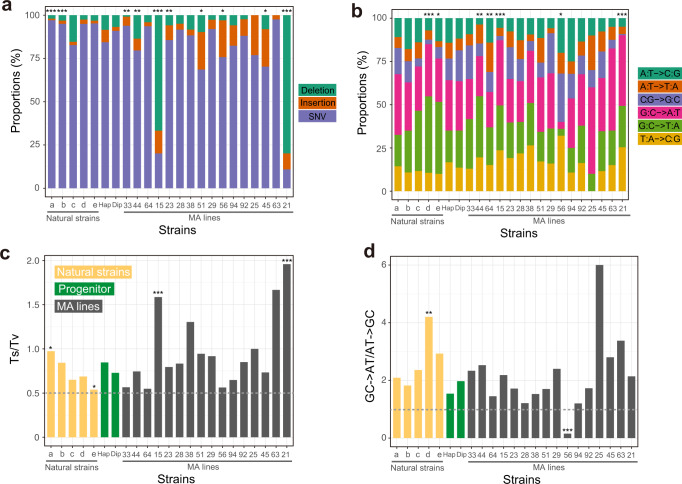


“**Fig. 4 Molecular spectra of mutations in 16 MA lines and 5 natural yeast strains estimated by MA + WGS.** **a** Proportions of SNVs, insertions, and deletions among all mutations. **b** Relative proportions of the six types of SNVs. **c** Number of transition mutations relative to the number of transversion mutations (Ts/Tv). **d** Number of GC→AT mutations relative to the number of AT→GC mutations. In **c** and **d**, the gray dotted line shows the random expectation. In all panels, the small letters on the X-axis refer to natural strains (a DBY4974/DBY4975; b SEY6211; c SK1/BY; d DBVPG6765; and e YPS128/DBVPG6765), “Hap” refers to the progenitor in the haploid form, “Dip” refers to the progenitor in the diploid form, and the numbers refer to IDs of MA lines. Two-tailed chi-squared test is performed between each strain and the progenitor: *, *P* *<* 0.05; **, *P* < 0.01; ***, *P* < 0.001.”

The previous incorrect version of Table 1:

**Table 1:** Test of stabilizing selection of the mutation rate in yeast.

	*V*_m_			*V*_mL_			*V*_mH_		
	*V*_m1_	*V*_m2_	*V*_m3_	*V*_mL1_	*V*_mL2_	*V*_mL3_	*V*_mH1_	*V*_mH2_	*V*_mH3_
*CAN1*-based tests (*V*_g_: 4.5 × 10^-2^)									
Mutational variance	5.4 × 10^-6^	9.7 × 10^-7^	3.5 × 10^-5^	4.4 × 10^-6^	7.9 × 10^-7^	2.8 × 10^-5^	9.5 × 10^-7^	1.7 × 10^-7^	6.1 × 10^-6^
*V*_g_*/V*_m_ (neutral expectation: 4 × 10^7^)	8.3 × 10^3 ¶^	4.6 × 10^4¶^	1.3 × 10^3¶^	1.0 × 10^4¶^	5.7 × 10^4¶^	1.6 × 10^3¶^	4.7 × 10^4 ¶^	2.6 × 10^5 ¶^	7.3 × 10^3¶^
MA+WGS-based tests (*D*^2^: 0.18)									
Mutational variance	3.6 × 10^-6^	6.5 × 10^-7^	2.3 × 10^-5^	1.3 × 10^-7^	2.4 × 10^-8^	8.5 × 10^-7^	2.2 × 10^-6^	4.0 × 10^-7^	1.5 × 10^-5^
*D*^*2*^*/V*_m_ (neutral expectation: 2.89 × 10^9^)	5.0 × 10^4 ¶^	2.8 × 10^5¶^	7.8 × 10^3¶^	1.4 × 10^6*^	7.7 × 10^6*^	2.2 × 10^5*^	8.2 × 10^4¶^	4.5 × 10^5¶^	1.3 × 10^4¶^

All mutation frequencies/rates are log_10_-transofrmed before the test. *CAN1*-based intraspecific mutation rate variance *V*_g_ is from 7 natural strains while *V*_m_ is from 48 MA lines. MA+WGS-based *D*^*2*^ is the squared difference in SNV mutation rate between *S. cerevisiae* and *S. paradoxus*, while *V*_m_ is based on the SNV mutation rates of 13 MA lines. All *V*_g_/*V*_m_ and *D*^2^/*V*_m_ ratios are significantly below the corresponding neutral expectations based on bootstrap tests (^*^P < 0.05; ^¶^*P* < 0.0001).

has been replaced with the correct version of Table 1

**Table 1:** Test of stabilizing selection of the mutation rate in yeast.

	*V*_m_			*V*_mL_			*V*_mH_		
	*V*_m1_	*V*_m2_	*V*_m3_	*V*_mL1_	*V*_mL2_	*V*_mL3_	*V*_mH1_	*V*_mH2_	*V*_mH3_
*CAN1*-based tests (*V*_g_: 4.5 × 10^-2^)									
*V*_mL_Mutational variance	3.4 × 10^-6^	6.1 × 10^-7^	2.2 × 10^-5^	1.9 × 10^-6^	3.4 × 10^-7^	1.2 × 10^-5^	1.4 × 10^-6^	2.6 × 10^-7^	9.2 × 10^-6^
*V*_mL_*V*_g_*/V*_m_ (neutral expectation: 4 × 10^7^)	1.3 × 10^4 ¶^	7.4 × 10^4¶^	2.0 × 10^3¶^	2.4 × 10^4¶^	1.3 × 10^5¶^	3.7 × 10^3¶^	3.1 × 10^4 ¶^	1.7 × 10^5 ¶^	4.9 × 10^3¶^
MA+WGS-based tests (*D*^2^: 0.18)									
Mutational variance	1.4 × 10^-6^	2.6 × 10^-7^	9.3 × 10^-6^	1.3 × 10^-7^	2.6 × 10^-8^	9.3 × 10^-7^	8.5 × 10^-7^	1.5 × 10^-7^	5.5 × 10^-6^
*V*_mL_*D*^*2*^*/V*_m_ (neutral expectation: 2.89 × 10^9^)	1.3 × 10^5 ¶^	6.9 × 10^5¶^	1.9 × 10^4¶^	1.3 × 10^6*^	7.0 × 10^6*^	1.9 × 10^5*^	2.1 × 10^5¶^	1.2 × 10^6¶^	3.3 × 10^4¶^

All mutation frequencies/rates are log_10_-transofrmed before the test. *CAN1*-based intraspecific mutation rate variance *V*_g_ is from 7 natural strains while *V*_m_ is from 44 haploid MA lines. MA+WGS-based *D*^*2*^ is the squared difference in SNV mutation rate between *S. cerevisiae* and *S. paradoxus*, while *V*_m_ is based on the SNV mutation rates of 16 MA lines. All *V*_g_/*V*_m_ and *D*^2^/*V*_m_ ratios are significantly below the corresponding neutral expectations based on bootstrap tests (^*^P < 0.001; ^¶^*P* < 0.0001).

- The original version of the Supplementary Information associated with this Article contained errors in Supplementary Figure 2 and Supplementary Tables 2, 4, 6, 7 and 8. The HTML has been updated to include a corrected version of the Supplementary Information; the original incorrect versions of this Figure and these Tables can be found as Supplementary Information associated with this Correction.
- The original version of the Supplementary Information associated with this Article included an incorrect Supplementary Data 1 file. The HTML has been updated to include a corrected version of Supplementary Data 1; the original incorrect version of Supplementary Data 1 can be found as Supplementary Information associated with this Correction.
- The original version of the Source Data associated with this Article contained errors reflecting the changes made to the data and figures described above. The HTML has been updated to include a corrected version of the Source Data; the original incorrect version of the Source Data can be found as Supplementary Information associated with this Correction.

## Supplementary information

Updated Supplementary Information

Incorrect Supplementary Information

Updated Supplementary Data 1

Incorrect Supplementary Data 1

Updated Source Data

Incorrect Source Data

