# Supplementary material for: The rate and molecular spectrum of mutation are selectively maintained in yeast
Source: Nat Commun. 2021 Jun 30;12:4044. doi: 10.1038/s41467-021-24364-6 (PMC8245649; doi:10.1038/s41467-021-24364-6)
Supplement: Supplementary file 1 — Supplementary Information [file 41467_2021_24364_MOESM1_ESM.pdf]

SUPPLEMENTARY MATERIALS FOR

The rate and molecular spectrum of mutation are selectively maintained in yeast

H. Liu & J. Zhang (jianzhi@umich.edu)

Supplementary materials include:
Supplementary Tables 1-8
Supplementary Figures 1-4

Supplementary Table 1. Summary statistics of yeast strains from the first round of mutation accumulation.

Sample	# of generations	Sequencing depth	# of SNVs	# of insertions	# of deletions	Total # of mutations	# of genes with mutations
Progenitor	NA	44.31×	NA	NA	NA	NA	NA
MA1	1435	78.27×	69	29	388	486	123
MA2	1528	71.74×	64	87	667	818	135
MA3	1535	67.25×	78	82	734	894	183
MA5	1515	135.73×	172	102	604	878	211
MA6	1452	47.34×	182	102	644	928	228
MA8	1498	46.71×	124	79	723	926	184
MA9	1528	78.35×	94	79	675	848	163
MA10	1544	104.64×	96	82	606	784	158
MA11	1475	64.34×	143	80	693	916	204
MA12	1510	29.99×	157	89	544	790	195
MA13	1520	93.15×	120	55	529	704	160
MA14	1544	130.88×	170	105	786	1061	236
MA15	1515	103.08×	113	90	745	948	190
MA16	1475	120.1×	66	81	659	806	152
MA17	1510	73.83×	88	62	759	909	178
MA19	1475	104.98×	107	93	715	915	187
MA20	1535	103.18×	109	104	756	969	197
MA21	1584	79.30×	98	84	773	955	184
MA22	1612	236.62×	110	81	709	900	175
MA23	1435	125.04×	140	72	352	564	154
MA24	1550	105.33×	178	103	805	1086	232
MA25	1504	88.51×	158	121	668	947	223
MA26	1555	63.60×	163	96	730	989	225
MA27	1452	105.70×	82	87	715	884	159
MA28	1535	92.06×	59	53	608	720	121
MA29	1603	105.16×	74	72	685	831	142
MA30	1464	230.78×	236	113	721	1070	291
MA31	1515	97.44×	110	102	776	988	202
MA32	1538	60.02×	249	119	834	1202	332
MA33	1484	63.25×	85	57	741	883	172
MA34	1547	84.34×	63	48	575	686	123
MA35	1492	60.22×	144	95	692	931	217
MA38	1535	91.65×	128	98	757	983	218
MA39	1464	236.20×	82	77	669	828	151
MA40	1544	92.81×	211	158	741	1110	278
MA41	1557	82.31×	108	78	696	882	197
MA42	1541	85.30×	105	77	707	889	160
MA43	1570	47.03×	158	110	727	995	240
MA44	1524	73.82×	109	102	750	961	191

MA45	1504	105.52×	130	111	731	972	216
MA46	1535	71.60×	96	106	715	917	174
MA47	1498	87.27×	99	84	674	857	164
MA48	1475	70.89×	115	113	700	928	205
MA49	1538	68.41×	192	114	689	995	258
MA50	1484	49.62×	126	85	640	851	177
MA51	1538	123.24×	62	67	640	769	138
MA52	1464	89.74×	92	69	722	883	173
MA53	1435	66.44×	106	66	645	817	167
MA54	1412	70.33×	136	102	722	960	217
MA55	1524	87.94×	52	43	509	604	119
MA56	1510	68.62×	93	56	696	845	166
MA57	1532	88.24×	72	73	760	905	162
MA58	1560	132.53×	119	91	806	1016	218
MA59	1560	163.17×	102	103	636	841	167
MA60	1484	72.40×	88	88	689	865	168
MA61	1475	175.62×	104	99	744	947	188
MA62	1452	109.87×	129	74	677	880	179
MA63	1550	76.51×	119	64	746	929	197
MA64	1412	62.29×	104	54	651	809	166
MA65	1498	233.19×	144	98	615	857	196
MA66	1550	25.49×	88	50	425	563	128
MA67	1484	67.76×	143	125	683	951	205
MA68	1544	126.37×	89	71	687	847	160
MA69	1412	98.77×	108	94	704	906	200
MA70	1372	45.61×	71	72	523	666	140
MA71	1492	60.37×	110	65	476	651	152
MA72	1524	65.11×	122	97	602	821	197
MA73	1538	186.73×	95	58	813	966	165
MA74	1528	143.57×	66	68	701	835	151
MA75	1412	95.13×	81	85	645	811	152
MA76	1544	122.19×	184	145	676	1005	250
MA77	1532	101.98×	99	92	711	902	174
MA78	1532	113.34×	70	85	704	859	147
MA79	1555	101.89×	85	96	716	897	160
MA80	1524	80.93×	69	57	664	790	139
MA81	1515	78.94×	75	75	678	828	161
MA82	1528	68.98×	81	102	668	851	152
MA83	1524	84.81×	189	113	690	992	257
MA85	1532	117.39×	114	80	690	884	191
MA86	1464	103.00×	166	92	746	1004	245
MA88	1524	99.84×	115	91	664	870	174
MA89	1552	96.14×	118	119	675	912	191
MA90	1535	36.86×	79	58	706	843	163
MA91	1535	44.68×	119	101	662	882	198

MA92	1541	100.94×	99	79	683	861	192
MA93	1555	66.62×	131	122	651	904	194
MA94	1552	85.92×	128	99	685	912	193
MA95	1372	86.80×	113	95	741	949	192
MA96	1498	181.65×	159	86	638	883	217
MA97	1515	56.27×	118	88	655	861	182
MA98	1504	94.26×	120	91	663	874	185
MA99	1557	178.49×	131	72	613	816	193
MA100	1520	65.48×	131	82	664	877	219

Supplementary Table 2. Summary statistics of yeast strains from the second round of mutation accumulation.

Strain	# of replicate lines	# of generations per line	# of SNVs*	# of insertions*	# of deletions*	Total # of mutations*
BY4741	20	1011.1	120	10	12	142
BY4743	20	1029.08	280	7	21	308
MA15	19	675.6	1368	886	4530	6784
MA21	4	665.5	142	120	1040	1302
MA23	17	638.5	131	13	9	153
MA25	3	499.7	10	3	0	13
MA28	19	868.7	55	2	3	60
MA29	8	692.9	69	4	2	75
MA33	20	719.6	108	7	0	115
MA38	20	970.2	53	5	2	60
MA44	20	637.0	82	7	14	103
MA45	4	496.8	26	8	3	37
MA51	3	508.4	35	11	5	51
MA56	4	512.9	25	7	1	33
MA63	1	712.6	40	1	0	41
MA64	20	491.6	206	5	9	220
MA92	4	688.6	37	4	1	42
MA94	3	507.2	28	4	2	34

*Combined from all replicate lines.

Supplementary Table 3. Origins and *CAN1* mutation frequencies of 7 natural yeast strains.

Strain	Mutation frequency*	Origin
CBS2888a	1.1×10^{-7}	Soil, South Africa
BY4724	1.7×10^{-7}	Lab derived
YJM454a	1.7×10^{-7}	Human, clinical
Clib219 (YST195)	1.7×10^{-7}	Wine, Russia
273614N (YST133)	2.2×10^{-7}	Human, clinical
I14	2.8×10^{-7}	Vineyard soil, Italy
RM11-1a	5.8×10^{-7}	Vineyard, U.S.

*Data from Gou *et al. Genetics* **211**, 731-740 (2019).

Supplementary Table 4. Test of stabilizing selection of the mutation rate in yeast.

	V_m			V_{mL}			V_{mH}		
	V_{m1}	V_{m2}	V_{m3}	V_{mL1}	V_{mL2}	V_{mL3}	V_{mH1}	V_{mH2}	V_{mH3}
***CAN1*-based tests** (V_g: 2.13×10^{-14})									
Mutational variance	4.7×10^{-17}	8.5×10^{-18}	3.1×10^{-16}	8.4×10^{-20}	1.5×10^{-20}	5.4×10^{-19}	6.2×10^{-17}	1.1×10^{-17}	4.0×10^{-16}
V_g/V_m (neutral expectation: 4×10^7)	4.5×10^{2} ¶	2.5×10^{3} ¶	7.0×10^{1} ¶	2.5×10^{5} ¶	1.4×10^{6} ¶	3.9×10^{4} ¶	3.5×10^{2} ¶	1.9×10^{3} ¶	5.4×10^{1} ¶
**MA+WGS-based tests** (D^2: 1.50×10^{-20})									
Mutational variance	2.2×10^{-23}	4.0×10^{-24}	1.4×10^{-22}	9.9×10^{-26}	1.8×10^{-26}	6.4×10^{-25}	2.4×10^{-23}	4.2×10^{-24}	1.5×10^{-22}
D^2/V_m (neutral expectation: 2.89×10^9)	6.7×10^{2} ¶	3.7×10^{3} ¶	1.0×10^{2} ¶	1.5×10^{5} *	8.5×10^{5} *	2.4×10^{4} *	6.4×10^{2} ¶	3.5×10^{3} ¶	9.8×10^{1} ¶

Same as Table 1 except that mutation frequencies/rates are not $\log_{10}$-transformed in the test. *CAN1*-based intraspecific mutation rate variance V_g is from 7 natural strains while V_m is from 44 haploid MA lines. MA+WGS-based D^2 is the squared difference in SNV mutation rate between *S. cerevisiae* and *S. paradoxus*, while V_m is based on the SNV mutation rates of 16 MA lines. All V_g/V_m and D^2/V_m ratios are significantly below the corresponding neutral expectations based on bootstrap tests (*, $P < 0.001$; ¶, $P < 0.0001$).

Supplementary Table 5. Top 27 candidate mutator genes.

Gene ID	Gene name	Mutator index*	Annotated function&
YDR217C	*RAD9*	0.44	DNA damage-dependent checkpoint protein
YFL013W-A		0.36	Dubious open reading frame
YML017W	*PSP2*	0.36	Polymerase SuPpressor
YDL036C	*PUS9*	0.31	Mitochondrial tRNA:pseudouridine synthase
YJL131C	*AIM23*	0.31	Mitochondrial translation initiation factor 3
YOR034C-A		0.31	Putative protein of unknown function
YDL034W		0.28	Dubious open reading frame
YJL182C		0.28	Dubious open reading frame
YJR119C	*JHD2*	0.28	JmjC domain family histone demethylase
YBR147W	*RTC2*	0.25	Putative vacuolar membrane transporter for cationic amino acids
YHR095W		0.25	Dubious open reading frame
YLR061W	*RPL22A*	0.25	Ribosomal 60S subunit protein L22A
YNR065C		0.25	Protein of unknown function
YOR304W	*ISW2*	0.25	ATP-dependent DNA translocase involved in chromatin remodeling
YOR219C	*STE13*	0.25	Dipeptidyl aminopeptidase
YJR094W-A	*RPL43B*	0.25	Ribosomal 60S subunit protein L43B
YFL039C	*ACT1*	0.22	Actin; structural protein involved in cell polarization
YNL067W-B		0.22	Putative protein of unknown function
YDR223W	*CRF1*	0.22	involved in repression of ribosomal protein (RP) gene transcription
YBL012C		0.22	Dubious open reading frame
YDL196W		0.22	Dubious open reading frame
YFL003C	*MSH4*	0.22	Protein involved in meiotic recombination
YJR050W	*ISY1*	0.22	Member of the NineTeen Complex (NTC)
YJR131W	*MNS1*	0.22	Alpha-1,2-mannosidase; involved in ER-associated protein degradation (ERAD)
YLR341W	*SPO77*	0.22	Meiosis-specific protein of unknown function
YNL012W	*SPO1*	0.22	Meiosis-specific prospore protein
YOR380W	*RDR1*	0.22	Transcriptional repressor involved in regulating multidrug resistance

*The mutator index of a gene is defined by $m_L/n_L - m_H/n_H$, where n_L is the number of MA lines with lower μ than the progenitor, m_L is the number of MA lines with lower μ than the progenitor and frameshift mutations in the focal gene, n_H is the number of MA lines with higher μ than the progenitor, and m_H is the number of MA lines with higher μ than the progenitor and frameshift mutations in the focal gene.

&Information retrieved from *Saccharomyces* genome database (www.yeastgenome.org).

Supplementary Table 6. Test of stabilizing selection of the yeast mutation spectrum.

Trait	V_g	V_{m1}	V_{m2}	V_{m3}	V_g/V_{m1}	V_g/V_{m2}	V_g/V_{m3}
SNV fraction	2.72×10^{-3}	4.95×10^{-7}	8.88×10^{-8}	3.19×10^{-6}	5.52×10^{3}	3.07×10^{4}	8.56×10^{2}
Insertion fraction	2.80×10^{-5}	4.37×10^{-8}	7.84×10^{-9}	2.82×10^{-7}	6.41×10^{2}	3.57×10^{3}	9.94×10^{1}
Deletion fraction	2.59×10^{-3}	4.41×10^{-7}	7.92×10^{-8}	2.85×10^{-6}	5.87×10^{3}	3.27×10^{4}	9.10×10^{2}
A:T→C:G fraction	1.26×10^{-3}	2.62×10^{-8}	4.70×10^{-9}	1.69×10^{-7}	4.82×10^{4}	2.68×10^{5}	7.47×10^{3}
A:T→T:A fraction	3.87×10^{-4}	1.83×10^{-8}	3.29×10^{-9}	1.18×10^{-7}	2.11×10^{4}	1.18×10^{5}	3.28×10^{3}
C:G→G:C fraction	2.21×10^{-3}	4.39×10^{-8}	7.88×10^{-9}	2.83×10^{-7}	5.04×10^{4}	2.81×10^{5}	7.81×10^{3}
G:C→A:T fraction	1.31×10^{-3}	9.48×10^{-8}	1.70×10^{-8}	6.11×10^{-7}	1.38×10^{4}	7.70×10^{4}	2.14×10^{3}
G:C→T:A fraction	1.00×10^{-2}	4.14×10^{-8}	7.43×10^{-9}	2.67×10^{-7}	2.42×10^{5}	1.35×10^{6}	3.75×10^{4}
T:A→C:G fraction	2.38×10^{-4}	4.37×10^{-8}	7.85×10^{-9}	2.82×10^{-7}	5.44×10^{3}	3.03×10^{4}	8.44×10^{2}
Ts/Tv	2.34×10^{-2}	1.43×10^{-6}	2.56×10^{-7}	9.21×10^{-5}	1.64×10^{4}	9.13×10^{4}	2.54×10^{3}
AT bias	0.711	1.26×10^{-5}	2.27×10^{-6}	8.14×10^{-5}	5.63×10^{4}	3.14×10^{5}	8.73×10^{3}

All V_g/V_{m1}, V_g/V_{m2}, and V_g/V_{m3} ratios are significantly below the neutral expectation of 4×10^{7} ($P < 0.001$ based on 10,000 bootstraps of natural strains as well as MA lines).

Supplementary Table 7. Test of stabilizing selection of the yeast mutation spectrum based on log_{10}-transformed trait values.

Trait	V_g	V_{m1}	V_{m2}	V_{m3}	V_g/V_{m1}	V_g/V_{m2}	V_g/V_{m3}
SNV fraction	$6.45×10^{-4}$	$5.53×10^{-7}$	$9.93×10^{-8}$	$3.57×10^{-6}$	$1.17×10^{3}$	$6.50×10^{3}$	$1.81×10^{2}$
Insertion fraction	$2.24×10^{-2}$	$9.24×10^{-7}$	$1.66×10^{-7}$	$5.96×10^{-6}$	$2.43×10^{4}$	$1.35×10^{5}$	$3.76×10^{3}$
Deletion fraction	$9.80×10^{-2}$	$2.16×10^{-6}$	$3.88×10^{-7}$	$1.39×10^{-5}$	$4.53×10^{4}$	$2.53×10^{5}$	$7.03×10^{3}$
A:T→C:G fraction	$1.80×10^{-2}$	$4.16×10^{-7}$	$7.47×10^{-8}$	$2.69×10^{-6}$	$4.32×10^{4}$	$2.41×10^{5}$	$6.70×10^{3}$
A:T→T:A fraction	$2.19×10^{-2}$	$4.50×10^{-7}$	$8.08×10^{-8}$	$2.90×10^{-6}$	$4.86×10^{4}$	$2.71×10^{5}$	$7.54×10^{3}$
C:G→G:C fraction	$7.61×10^{-2}$	$1.26×10^{-6}$	$2.27×10^{-7}$	$8.15×10^{-6}$	$6.03×10^{4}$	$3.36×10^{5}$	$9.34×10^{3}$
G:C→A:T fraction	$2.81×10^{-3}$	$4.79×10^{-7}$	$8.60×10^{-8}$	$3.09×10^{-6}$	$5.87×10^{3}$	$3.27×10^{4}$	$9.10×10^{2}$
G:C→T:A fraction	$2.16×10^{-2}$	$3.73×10^{-7}$	$6.70×10^{-8}$	$2.41×10^{-6}$	$5.79×10^{4}$	$3.23×10^{5}$	$8.98×10^{3}$
T:A→C:G fraction	$3.02×10^{-3}$	$1.45×10^{-7}$	$2.61×10^{-8}$	$9.37×10^{-7}$	$2.08×10^{4}$	$1.16×10^{5}$	$3.22×10^{3}$
Ts/Tv	$8.06×10^{-3}$	$2.31×10^{-7}$	$4.15×10^{-8}$	$1.49×10^{-6}$	$3.49×10^{4}$	$1.49×10^{5}$	$5.41×10^{3}$
AT bias	$1.61×10^{-2}$	$8.68×10^{-7}$	$1.56×10^{-7}$	$5.60×10^{-6}$	$1.86×10^{4}$	$1.03×10^{5}$	$2.88×10^{3}$

Same as Supplementary Table 6 except that all trait values are log_{10}-trasformed for computing V_g and V_m. All V_g/V_{m1}, V_g/V_{m2}, and V_g/V_{m3} ratios are significantly below the neutral expectation of $4×10^7$ ($P < 0.0001$ based on 10,000 bootstraps of natural strains as well as MA lines).

Supplementary Table 8. List of primers used in this study.

Name	Sequence	Annotation
**MSH2_ repair_U1**	TCT GCT GAC CTA ACA TCA AAA T	Used to synthesize the repair fragment that used for the reinsertion of the whole CDS of MSH2 (Forward primer)
**MSH2_ repair_L1**	CTA TCG ATT CTC ACT TAA GAT G	Used to synthesize the repair fragment that used for the reinsertion of the whole CDS of MSH2 (Reverse primer)
**MSH2_ repair_U2**	AAT CAG AAC TCC AGC ACT CCG	Used to synthesize the repair fragment that used for the reinsertion of the whole CDS of MSH2 (Forward primer)
**MSH2_ repair_L2**	CCC ATC CAT AGT TCT GAT ACC G	Used to synthesize the repair fragment that used for the reinsertion of the whole CDS of MSH2 (Reverse primer)
**RAD9_ repair_U**	tATTTAATCGTCCCTTTCTATCAATTATGAG TTTATATATTTTTATAATTAGCCCTGATG	Used to synthesize the repair fragment that used to knockout the whole CDS of RAD9 (Forward primer)
**RAD9_ repair_L**	AGAAACGCCATAGAAAAGAGCATAGTGA GAAAATCTTCAACATCAGGGCTAATTATA AAA	Used to synthesize the repair fragment that used to knockout the whole CDS of RAD9 (Reverse primer)
**YFL013W_ repair_U**	TTTAAAAATTAGCTGTTTTTTTaaaaaaaaaaa TTCTCTTTCTTTACAAACTGCGTTCCG	Used to synthesize the repair fragment that used to knockout the whole CDS of YFL013W-A (Forward primer)
**YFL013W_ repair_L**	GAAGATGGTGCACCTGCTGGAAAGTTCA TTTTAAGGTGTACGGAACGCAGTTTGTAA AGA	Used to synthesize the repair fragment that used to knockout the whole CDS of YFL-13W-A (Reverse primer)
**PSP2_ repair_U**	CACGTTTGCTCACTCGATCTTAATCACAT AGAGTGCTGGAACGGGAAGAAGCGGTA ACTA	Used to synthesize the repair fragment that used to knockout the whole CDS of PSP2 (Forward primer)
**PSP2_ repair_L**	TCATAAAGGCATGTCTGTTGTTCTGTTATT GTAGTTGGAGTAGTTACCGCTTCTTCCCG T	Used to synthesize the repair fragment that used to knockout the whole CDS of PSP2 (Reverse primer)
**MSH4_ repair_U**	TCTGTACAGAAATAATGGATTATAGTTTTA AGCTAAGCGGAAAAGCCAAATGCATATA GT	Used to synthesize the repair fragment that used to knockout the whole CDS of MSH4 (Forward primer)

**MSH4_ repair_L**	AACTAGTTATAGCATTGAAATCTGTAGCT GATCAACGCAAACTATATGCATTTGGCTT TT	Used to synthesize the repair fragment that used to knockout the whole CDS of MSH4 (Reverse primer)
**MSH2_ gRNA_U1**	GAT CAA ACA GGA ATC GAA TGC AAC GTT TTA GAG CTA G	Used to synthesize the gRNA that used for the reinsertion of MSH2 (Forward primer)
**MSH2_ gRNA_L1**	CTA GCT CTA AAA CGT TGC ATT CGA TTC CTG TTT	Used to synthesize the gRNA that used for the reinsertion of MSH2 (Reverse primer)
**MSH2_ gRNA_U2**	GAT CAC GAC TGA ATC CGG TGA GAA GTT TTA GAG CTA G	Used to synthesize the gRNA that used for the reinsertion of MSH2 (Forward primer)
**MSH2_ gRNA_L2**	CTA GCT CTA AAA CTT CTC ACC GGA TTC AGT CGT	Used to synthesize the gRNA that used for the reinsertion of MSH2 (Reverse primer)
**RAD9_ gRNA_U**	GATCCAGACCATTGAATCGCAAGGGTTTT AGAGCTAG	Used to synthesize the gRNA that target the gene RAD9 (Forward primer)
**RAD9_ gRNA_L**	CTAGCTCTAAAACCCTTGCGATTCAATGG TCTG	Used to synthesize the gRNA that target the gene RAD9 (Reverse primer)
**YFL013W-A_gRNA_U**	GATCTCACTCCAGCTTAAACATGGGTTTT AGAGCTAG	Used to synthesize the gRNA that target the gene YFL013W-A (Forward primer)
**YFL013W-A_gRNA_L**	CTAGCTCTAAAACCCATGTTTAAGCTGGA GTGA	Used to synthesize the gRNA that target the gene YFL013W-A (Reverse primer)
**PSP2_ gRNA_U**	GATCTGACATCTTGAAACAAACCAGTTTT AGAGCTAG	Used to synthesize the gRNA that target the gene PSP2 (Forward primer)
**PSP2_ gRNA_L**	CTAGCTCTAAAACTGGTTTGTTTCAAGAT GTCA	Used to synthesize the gRNA that target the gene PSP2 (Reverse primer)
**MSH4_ gRNA_U**	GATCTGCCAAGAGACATAAGTACGGTTTT AGAGCTAG	Used to synthesize the gRNA that target the gene MSH4 (Forward primer)
**MSH4_ gRNA_L**	CTAGCTCTAAAACCGTACTTATGTCTCTT GGCA	Used to synthesize the gRNA that target the gene MSH4 (Reverse primer)
**Psp2_Part1_ Repair_U**	GTTTGCTCACTCGATCTTAATCACATAGA GTGCTGGAACGGGAAGAAATGTCATTAA TCC	Used to synthesize the repair fragment that used to knockout the part 1 of PSP2 (Forward primer)
**Psp2_Part1_ Repair_L**	TTGCTGCCTGTTGCAGTGGTTGCCATAGA ATCACTACTAGGGATTAATGACATTTCTTC	Used to synthesize the repair fragment that used to knockout

	C	the part 1 of PSP2 (Reverse primer)
**Psp2_Part2_Repair_U**	AAGAAAAAATGGAAAATTTACACGTGGAAGATACAACAACCCTGAGGGCAAGAGGGAGTA	Used to synthesize the repair fragment that used to knockout the part 2 of PSP2 (Forward primer)
**Psp2_Part2_Repair_L**	CCTTTATAACTGCTGCCATTGGGGCCTCCACGATATCTATTACTCCCTCTTGCCCTCAGG	Used to synthesize the repair fragment that used to knockout the part 2 of PSP2 (Reverse primer)
**Psp2_Part3_Repair_U**	TGAGCAGAACCAAATATAACGGAAACCATAATAACAATAATGGCAATTTTAATAACAGAA	Used to synthesize the repair fragment that used to knockout the part 3 of PSP2 (Forward primer)
**Psp2_Part3_Repair_L**	TTAATGGGTCAGTACATGACTCATAAAGGCATGTCTGTTGTTCTGTTATTAAAATTGCCA	Used to synthesize the repair fragment that used to knockout the part 3 of PSP2 (Reverse primer)
**Psp2_Part2_gRNA_U**	GATCAAATCGGACGAGTTCAAAGGGTTTTAGAGCTAG	Used to synthesize the gRNA that target the part 2 of gene PSP2 (Forward primer)
**Psp2_Part2_gRNA_L**	CTAGCTCTAAAACCCTTTGAACTCGTCCGATTT	Used to synthesize the gRNA that target the part 2 of gene PSP2 (Reverse primer)
**Psp2_Part3_gRNA_U**	GATCAATCGCGGCGGATATCGTGGGTTTTAGAGCTAG	Used to synthesize the gRNA that target the part 3 of gene PSP2 (Forward primer)
**Psp2_Part3_gRNA_L**	CTAGCTCTAAAACCCACGATATCCGCCGCGATT	Used to synthesize the gRNA that target the part 3 of gene PSP2 (Reverse primer)
**Mutation_confirmation_1U**	CGCGCGCGTTTGGTAAGTAGG	Used for PCR of genomic positions that carry mutations
**Mutation_confirmation_2U**	AGCGCTGTCACTGCTACGACA	Used for PCR of genomic positions that carry mutations
**Mutation_confirmation_3U**	TGCAGAGCGTGTCGGCGTAC	Used for PCR of genomic positions that carry mutations
**Mutation_confirmation_4U**	ACGGCTGCTCTACACCTATGTCGT	Used for PCR of genomic positions that carry mutations
**Mutation_confirmation_5U**	TGCCAGCTGCTACCCAGGGA	Used for PCR of genomic positions that carry mutations
**Mutation_co**	TGGCAAACGCGGGGGAGAAG	Used for PCR of genomic

**nfirmation_6U**		positions that carry mutations
**Mutation_confirmation_7U**	CCGCCCCACGTGCTGGAAAT	Used for PCR of genomic positions that carry mutations
**Mutation_confirmation_8U**	ATGCGGTGCTGTGCTGGCTT	Used for PCR of genomic positions that carry mutations
**Mutation_confirmation_9U**	AGCCCCGAAGAACAACCGAGG	Used for PCR of genomic positions that carry mutations
**Mutation_confirmation_10U**	TGCCGACGTTCCAGTCAAGGA	Used for PCR of genomic positions that carry mutations
**Mutation_confirmation_1L**	AAGTCAGGCAGCCGTCCCCT	Used for PCR of genomic positions that carry mutations
**Mutation_confirmation_2L**	ACCTCATGCGGCGCTACTGA	Used for PCR of genomic positions that carry mutations
**Mutation_confirmation_3L**	TCATGCCCCCGTTACACGCC	Used for PCR of genomic positions that carry mutations
**Mutation_confirmation_4L**	CATAGTCGCGCGCCCTTGGT	Used for PCR of genomic positions that carry mutations
**Mutation_confirmation_5L**	CGATGGCCGCACTCACACCA	Used for PCR of genomic positions that carry mutations
**Mutation_confirmation_6L**	TCGGGCAATCCAAGAGCGCC	Used for PCR of genomic positions that carry mutations
**Mutation_confirmation_7L**	GGACCGGCCCATCAGAAGCA	Used for PCR of genomic positions that carry mutations
**Mutation_confirmation_8L**	CGGTTCTGAAGCGGCTTCTTGT	Used for PCR of genomic positions that carry mutations
**Mutation_confirmation_9L**	ACCCATTCCTCCATGGCAAC	Used for PCR of genomic positions that carry mutations
**Mutation_confirmation_10L**	TCGTCATAACCCGTTCAGTTCCT	Used for PCR of genomic positions that carry mutations

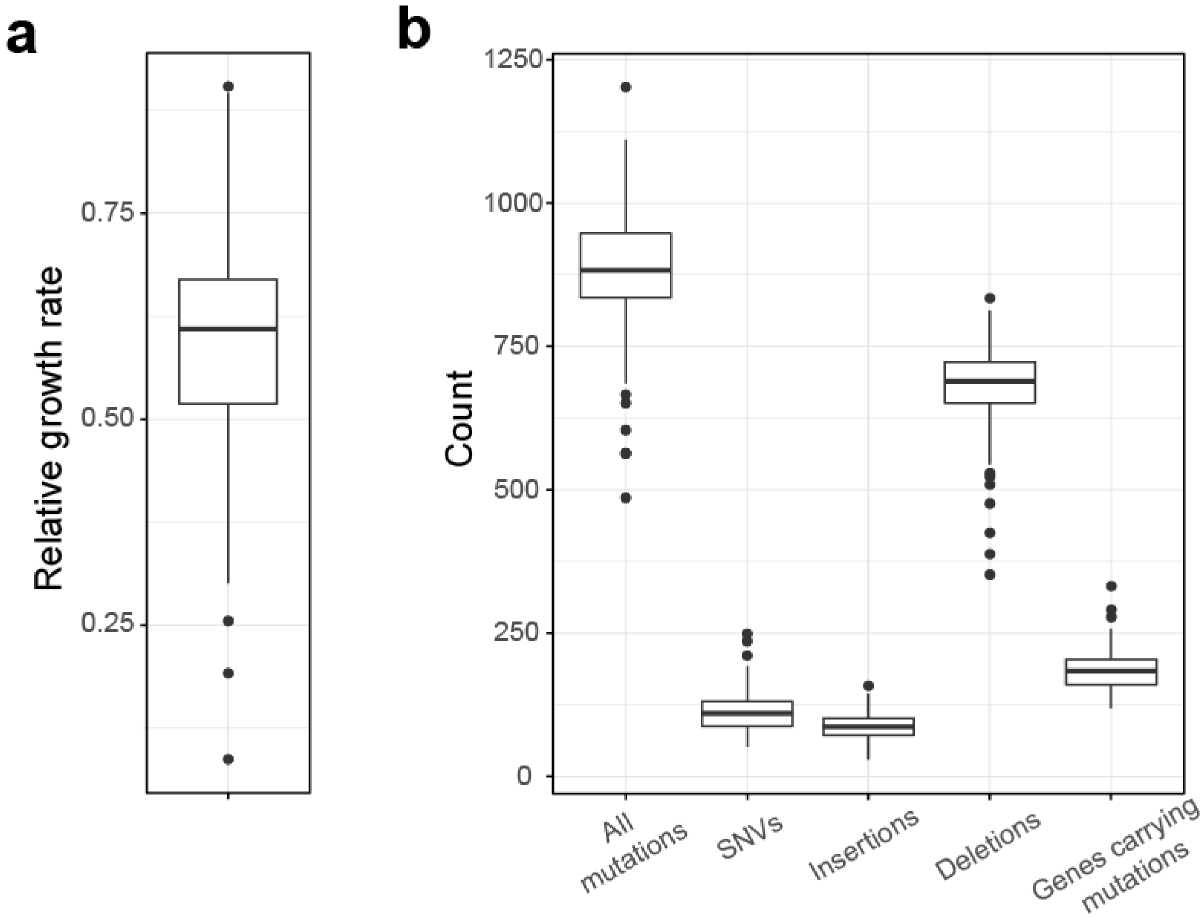

Supplementary Fig. 1. Growth rates of and mutations accumulated in the MA lines. a, Distribution of the growth rates of the 93 MA lines relative to that of the progenitor in liquid YPD medium. Growth rates of all strains, including the progenitor, were measured in the *MSH2*-lacking background. **b**, Distributions of numbers of various mutations and number of genes carrying mutations among the 93 MA lines. In each box plot, the lower and upper edges of a box represent the first (qu1) and third quartiles (qu3), respectively, and the horizontal line inside the box indicates the median (md). The whiskers extend to the most extreme values inside inner fences, md ± 1.5(qu3–qu1), and the dots represent values outside the inner fences (outliers).

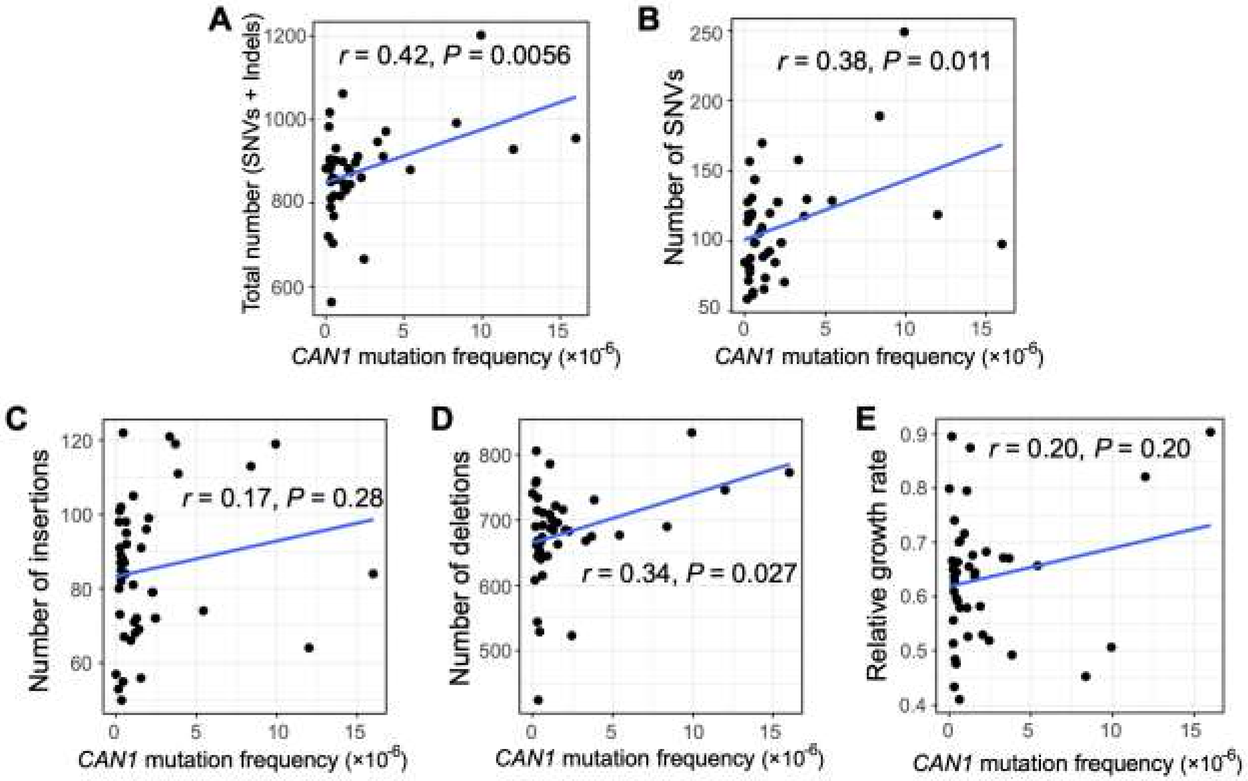

Supplementary Fig. 2. The *CAN1* mutation frequency of an MA line is significantly correlated with the number of mutations accumulated in the MA line but is not significantly correlated with its relative growth rate. a-d, Correlation between the *CAN1* mutation frequency and the total number of SNVs and indels (**a**), number of SNVs (**b**), number of insertions (**c**), or number of deletions (**d**) accumulated among 44 haploid MA lines. **e**, Correlation between the *CAN1* mutation frequency of an MA line and its growth rate relative to the progenitor among 44 haploid MA lines. In all panels, each dot represents one MA line and the blue line is the linear regression. Pearson's correlation coefficient (*r*) and associated *P*-value are presented. Growth rates were all measured in liquid YPD medium in the *MSH2*-lacking background.

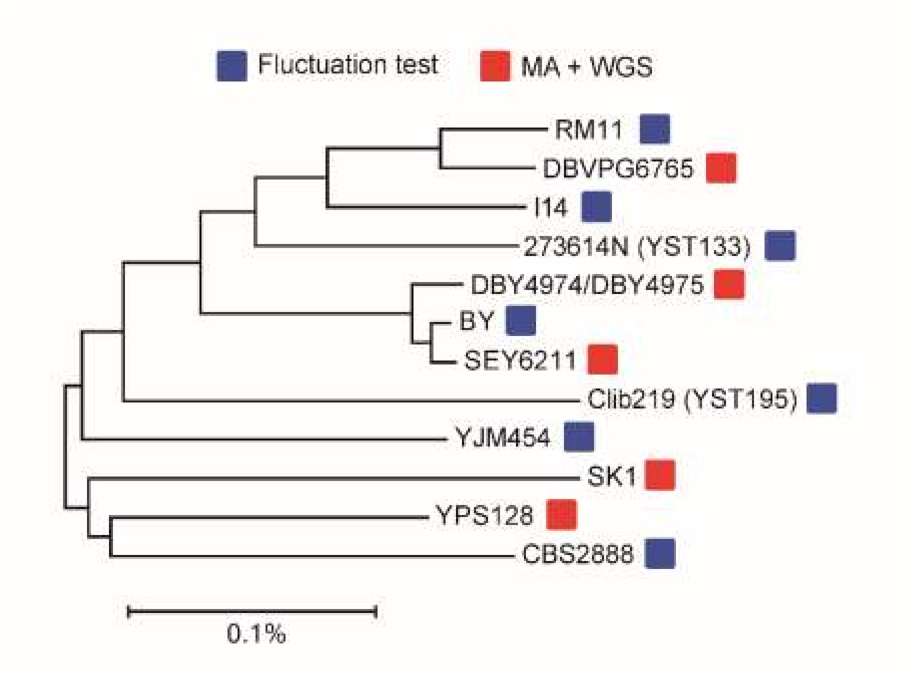

Supplementary Fig. 3. Evolutionary relationships among the natural yeast strains considered in this study. The tree was reconstructed by the neighbor-joining method with *p*-distance based on genome-wide SNVs. Strains with available fluctuation test-based *CAN1* mutation frequencies are marked by blue squares, whereas strains with MA+WGS-based estimates of mutation rate and spectrum are marked by red squares. The average genetic distance among the 7 strains marked in blue is 0.31% and that among the 5 strains marked in red is 0.30%. The progenitor for MA is derived from BY.

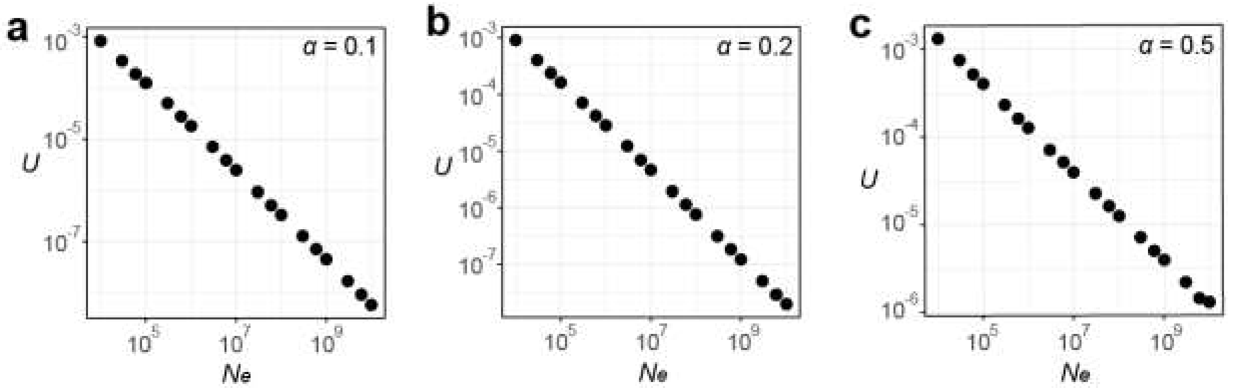

Supplementary Fig 4. The optimal mutation rate per functional genome per generation (*U*) declines with the effective population size (N_e) under the two second-order selections. Fitness disadvantages of deleterious mutations follow a gamma distribution with the mean equal to 0.01. Three gamma distributions with different shape parameters (α) are considered in the three panels, respectively.
