# Supplementary material for: The rate and molecular spectrum of mutation are selectively maintained in yeast
Source: Nat Commun. 2021 Jun 30;12:4044. doi: 10.1038/s41467-021-24364-6 (PMC8245649; doi:10.1038/s41467-021-24364-6)
Supplement: Supplementary file 4 — Description of Additional Supplementary Files [file 41467_2021_24364_MOESM4_ESM.pdf]

Description of Additional Supplementary Files

File Name: Supplementary Data 1
Description: Mutations identified in two rounds of mutation accumulation and CAN1 mutation frequencies.
